# Transient Visual Loss in a Hepatitis C Patient Treated With Pegylated Interferon Alfa-2a and Ribavirin

**DOI:** 10.5812/hepatmon.15124

**Published:** 2014-02-20

**Authors:** Ligita Jancoriene, Dovile Norvydaite, Saulius Galgauskas, Evelina Balciunaite

**Affiliations:** 1 Department of Infectious, Chest Diseases, Dermatovenerology and Alergology, Faculty of Medicine, Vilnius University, Vilnius, Lithuania; 2 Department of Otolaryngology and Eye Diseases, Faculty of Medicine, Vilnius University, Vilnius, Lithuania; 3 Department of xx, Faculty of Medicine, Vilnius University, Vilnius, Lithuania

**Keywords:** Hepatitis C, Chronic, Peginterferon alfa-2a, Optic Nerve Diseases, Pulse Therapy, Drug

## Abstract

**Introduction:**

Patients with Hepatitis C are commonly treated with combination of Pegylated Interferon alfa-2a and Ribavirin. Less than 1% of patients receiving this treatment experience very uncommon ophthalmological side effects such as optic neuropathy and vision disorder, which are usually subclinical, mild and reversible, not requiring the withdrawal of the treatment. Retinopathy is the most commonly reported ocular side effect of interferon use, usually presenting with cotton wool spots and retinal hemorrhages.

**Case Presentation:**

We represent a case of severe retinopathy and optic neuropathy in a patient with chronic hepatitis C genotype 3a infection, treated with the combination of PEG-IFN alfa-2a (180 mkg once weekly) and Ribavirin (1200 mg daily). Bilateral visual loss of both eyes developed at 11th week of therapy and changes in retina and optic nerve were observed. Fluorescein angiography and optical coherence tomography showed bilateral anterior ischemic optic neuropathy and macular edema. Visual acuity improved 1 month and fundoscopic changes were no longer present 6 months after the urgent permanent discontinuation of PEG-IFN treatment and the pulse steroid therapy followed by a 2 week course of oral prednisone.

**Discussion:**

In case of interferon-associated retinopathy discontinuation of the therapy and treatment with high dose steroids can be beneficial. The prognosis of interferon-associated opthalmological side effects remains uncertain: in some patients visual acuity improves, other continues with poor visual outcome. Considering that, all patients should undergo ophthalmologic examination before treatment with interferon and their ophthalmological status should be monitored regularly while receiving this therapy.

## 1. Introduction

Interferon is a group of proteins that has antiviral, antiproliferative and immunomodulating effects. During pegylation a polyethylene glycol molecule is attached to a protein in order to increase its molecular weight and the half-life of serum elimination. This process increases the antiviral effect and enables to use interferon once weekly, but a number of side effects increases too.

Interferon alfa (IFNalfa) has been widely used for the treatment of chronic viral hepatitis. The common side effects associated with this drug are well known. They include flu-like symptoms such as mild fever and chills, fatigue, myalgia, nausea, loss of appetite and psychiatric symptoms such as depression, suicidal ideation, irritability, nervousness and insomnia. Less common reported side effects are hematopoietic suppression, reversible hair loss, hearing loss, dermatitis, seizures, the development or exacerbation of autoimmune diseases such as thyroid dysfunction, rheumatoid arthritis, arteritis, cryoglobulinemia, sarcoidosis, systemic lupus erythematosus, vitiligo, type 1 diabetes and myasthenia gravis (1). Ophthalmological complications have been infrequently (< 1%) reported as a potentially serious adverse events (2). Known ocular side effects include hemorrhagic retinopathy with soft exudates in retina and atypical complications such as swelling of the optic nerve disc, microaneurysms, neovascular glaucoma, subconjunctival hemorrhage, dysfunction of retinal and choroidal perfusion, cystoid macular edema, retinal detachment, vitreous hemorrhage, panophthalmitis and oculomotor nerve paralysis. Optic neuropathy and vision disorders are relatively rare. Ocular complications are usually subclinical, mild and reversible, not requiring the withdrawal of the treatment (3). Occasionally cotton wool spots and retinal hemorrhages may affect visual acuity because of macular lesions. Retinopathy is the most commonly reported side effect of IFN use. However, Alteroche et al. have described neuro-visual impairment as the most common side effect in their study at 2006. 

The treatment approach consists mostly of hydration, prevention of hypotension and discontinuation of IFN alfa therapy. Pulse steroid application is another option, which was chosen in our case.

Recently some cases of optic neuropathy with visual impairment and some other peripheral neuropathies, including sensory and autonomic neuropathies, chronic inflammatory demyelinating polyneuropathy and Bell's palsy have been reported in patients treated with IFN alfa. We represent a case of severe retinopathy and neuropathy at 11th week of therapy developed in chronic hepatitis C (CHC) patient treated with the combination of pegylated interferon (PEG-IFN) alfa-2a and ribavirin.

## 2. Case Presentation

A 49 year-old man since 2007 was diagnosed with CHC genotype 3a infection, probably received during blood transfusion on 1989 due to the bleeding from stomach ulcer. Patient was slightly overweight (body mass index-27), had not adequately controlled primary arterial hypertension and farsightedness, acquired in childhood. Liver biopsy, performed on 2007, revealed mild liver inflammation, moderate hepatosteatosis and second stage of fibrosis according to METAVIR classification. Since 29th of June 2007 he started CHC treatment with PEG-IFN alfa-2a (Pegasys, F.Hoffmann- LaRoche) (180 mkg once weekly) and ribavirin (1200 mg daily). After the 11th injection of PEG-IFN alfa-2a sudden bilateral visual loss of both eyes developed and changes in retina and optic nerve were observed. It was found out, that the first transitory visual loss patient noticed after the 10th injection of PEG-IFN alfa-2a. At that time hypertensive retinopathy was suspected, because the patient used his antihypertensive medications irregularly.

**Figure 1.**
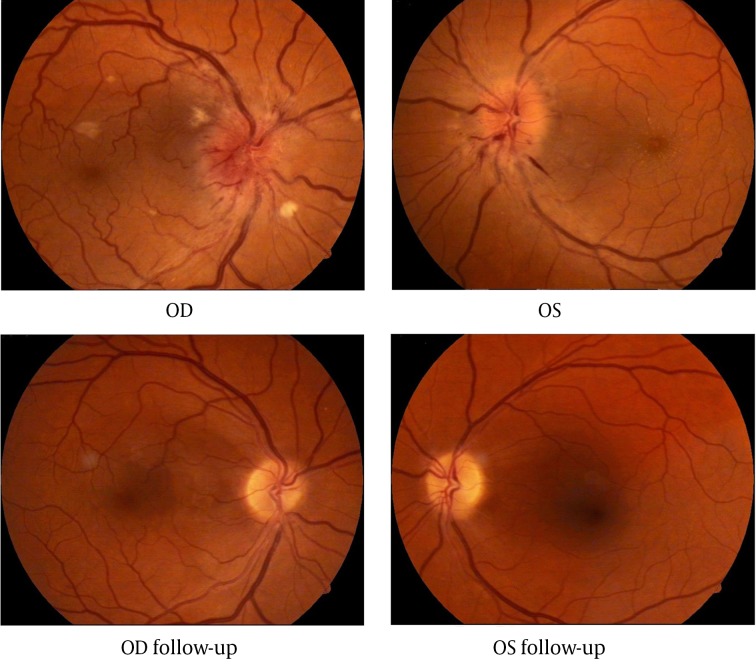
Fundus of Right and Left Eyes at the Time of Admitting and Five Years Later

At the time of admitting to the hospital patient’s visual acuity was 0.1 (with correction) in the right eye and 0.01 (incorrigible) in the left eye. The intraocular pressure in both eyes was 14 mmHg. Extra ocular motility was full. The pupillary reactions were normal. Fundus examination of both eyes revealed: swollen optic nerve disc with varicose vessels and blurred margins, linear radial hemorrhages and cotton wool spots (right eye more than left) near the edge of the optic disc, narrow arteries and varicose veins (Figure 1). Fluorescein angiography confirmed the bilateral anterior ischemic optic neuropathy. Macular edema was clearly observed with optical coherence tomography (OCT). Retinal thickness at 3 and 9 o’clock position was respectively 329/322 µm. 

Laboratory tests: serum transaminases, blood clotting parameters (partial thromboplastin time (PTT), international normalized ratio (INR)), basic blood test and urine test were normal: serum glucose 4.9 mmol/L, hematocrit 43%, hemoglobin 147 g/L, erythrocyte sedimentation rate 18mm/h, total bilirubin 8,5 mkmol/L, alkaline phosphatase 102 U/L, fibrinogen 4,87 g/L, D-dimmers 310 mkg/L.

Computed tomography (CT) and magnetic resonance imaging (MRI) of the head were performed, but no pathological findings were found and multiple sclerosis was ruled out.

Antiviral treatment was permanently discontinued. Treatment with intravenous methylprednisolone at a dose of 1 g/day for 3 days followed by a 2 week course of oral prednisone at a dose of 1 mg/kg/day tapering over 3 days. 

After the pulse therapy visual acuity increased from 0.1/0.01 to 0, 5/0, 2 and after one month (with optical correction) it was 0.8/0.7. 6 months later optic disc edema, hemorrhages and soft exudates were no longer visible. After 6 months OCT showed retinal thickness decreased to 245/224 μm at the same locations.

After 10 more months patients’ visual acuity was the same and no changes in retina were observed. OCT showed similar macular thickness, but the retinal nerve fiber layer was thinner on the temporal sides and the average thickness was 76.9/67.1 μm – below normal, which means sub atrophy of the optic nerve.

Patient was tested for HCV-RNA after the permanent discontinuation of antiviral CHC treatment, after 6 months follow-up and on May 2012 (5 years later) and, due to stabile negativity of HCV-RNA, sustained viral response was confirmed.

Ophthalmic examination after 5 years showed a visual acuity of 0.8 (with correction) for the right eye and 0.9 (with correction) for the left eye. Fundoscopy of both eyes was normal (Figure 1). 

## 3. Discussion

According to the literature, the incidence of retinopathy during interferon therapy is 18 - 86%, but most patients, who develop IFN-associated retinopathy, stay asymptomatic. Because of reversible retinal changes, subjective visual impairment and documented reduced visual acuities are rare. Diabetes mellitus, hypertension, anemia, thrombocytopenia and increased triglyceride levels are risk factors of interferon-associated retinopa-thy (4). Etiopathogenesis of interferon-induced vision changes is not clear. There are few theories. Ischemic: interferon activates endothelial growth factors and stimulates growth of high permeability microvessels followed by swelling of the surrounding tissue (5). This can also cause optic nerve ischemia and fiber swelling, progress to retinal vein occlusion and hemorrhage, anterior ischemic optic neuropathy with permanent losses of visual field and visual acuity (6). Immune: retinal micro vessels are blocked by immune complexes activating the complement system, which stimulates intravascular granulocyte aggregation. As the result, capillary infarctions and micro vessel embolization begin and cotton wool spots appear (7). So, HCV infection by itself may play a role in activating the clotting system by creating immune-mediated complexes consisting of HCV autoantibodies and HCV virions (8). High circulating levels of plasma-activated complement 5, which is a potent intravascular aggregator of platelets, favoring the development of microthrombi, were also found in patients treated with IFN alfa (7). It is likely that the levels of pro-inflammatory cytokines may trigger autoimmune phenomena in immunologically predisposed individuals when IFN is administered. The immune system mistakenly attacks the host's nerve tissue after recognizing a molecular epitope similar to a foreign antigen and this may result in acute inflammatory neuropathy. In addition, IFN alfa can enhance autoantibody production and may upregulate transcription of genes associated with class I major histocompatibility complex antigens. Nocturnal arterial hypotension could also be a part of the mechanism of the disease. IFN alfa causes systemic hypotension, and the resultant blood pressure fluctuations may induce vascular ischemia of the optic nerve (8). Patients with diabetes and primary arterial hypertension already have microcirculation disorders, so retinal side effects of interferon should appear more often. 

Interferon-associated retinopathy usually presents with cotton wool spots and retinal hemorrhages, most notably around the optic nerve head and in the posterior pole (9). It most frequently presents 4 to 12 weeks after treatment begins (10). These ocular findings appear to reverse with cessation of treatment. There is some evidence that the incidence of the retinopathy may be dose dependent. Hayasaka et al. at 1995 reported possible increased incidence in patients on higher and more frequent doses. Manesis et al. at 1998 also established that approximately 1 of 4 patients is expected to develop subclinical visual neurophysiologic abnormalities and a reduction in sensitivity in central vision. Older age and hypercholesterolemia were the main predictors of these abnormalities.

Several studies have been performed to evaluate and document the incidence of IFN-associated retinopathy. Cuthbertson et al. reported evidence of retinopathy consisting of cotton wool spots and/or hemorrhages in 4 of 25 patients (16%) after 3 months of treatment with PEG-IFN alfa and ribavirin. None had visual symptoms. Changes disappeared in all patients without any dosage alteration (11). This suggests that treatment can be continued in the presence of retinopathy. Chisholm et al. reported 9 of 10 patients, who received PEG-IFNalfa and ribavirin, having either abnormal retinal function or retinal changes on fundoscopy. No changes in visual acuity were noted (10). Mousa et al. analyzed 98 patients with CHC who underwent combination therapy of PEG-IFNalfa and ribavirin. Only 8 patients (8.16%) developed retinopathy (2 of them had diabetes, 1 had hypertension, 4 had both) (12).

We reviewed 10 cases of optic neuropathy in patients treated with IFN alfa (Table 1). It can occur any time after the start of interferon therapy and is potentially serious adverse event with probable severe visual disturbances. Usually it starts as sudden and painless vision loss in varying degrees. In most cases visual field defects are present. Color vision can be affected too. Because of its severe manifestation, optic neuropathy requires the withdrawal of the IFN therapy.

It is assumed that fundoscopy is most useful for determining the presence of cotton wool spots and retinal hemorrhage and perimetry for identifying visual field losses. There are also suggestions to use the focal electroretinogram or the Humphrey 10-2 visual field testing as more sensitive methods of testing for occult ischemic retinal damage (9).

Similar ocular side effects develop while using IFN alfa for treating other conditions. Interferon-associated anterior ischemic optic neuropathy with severe visual losses has been reported during treatment of malignant melanoma, essential thrombocytosis (13), kidney cancer (14), multiple myeloma, polycythemia vera, amyotrophic lateral sclerosis. Complications observed in those on high-dose IFN therapy for tumor treatment are usually more severe (10).

Our patient received steroids with favorable course of visual function. His symptoms improved 1 month after the urgent permanent discontinuation of PEG-IFN treatment and the pulse steroid therapy. In most our reviewed cases visual disturbances haven’t recovered completely. The prognosis of interferon-associated optic neuropathy is uncertain. In some patients visual acuity improves, others continue with poor visual outcome despite discontinuation of the IFNalfa and additional treatments. Some authors recommend regular ophthalmologist consultations for all patients treated with interferon (8, 14), others, however, claim that it is not necessary for those who have no vision problems (11).

**Table 1.** Cases of Interferon-Associated Optic Neuropathy

	HCV therapy	Interval before first ocular manifestations	Symptoms	Vision	Fundoscopy	Other	Therapeutic management	Further course
Age, Sex								
60 year old woman (15)	Various types of IFN, at the end PEG-IFNα and RBV ^a^	OS a 10 months, OD a 17 months	Pain when moving left eye, visual loss	OU 0.02 (light perception and finger counting). Visual field defects	n.d.	No response to visual evoked potentials OU	IFN termined, triamcinolone injection, MTX a, IVMP a (4 courses), IVIg (5 days), CPM a (1 day), plasmapheresis (10 courses)	The exacerbation of visual impairment was halted
38 year old man (7)	PEG-IFNα (180 µg/week) and RBV (800 mg/day)	n.d.	Sudden painless blurring of vision and progressing black shadow in OS	OS 0.05, OD 0.1. Impairment of color vision OS. Visual field (inferior altitudinal) defect OS	OS: optic disc elevated and edematous with tortuous dilated vessels, splinter hemorrhages, cotton wool spots. OD: crowded disc, pallid edema of the optic disc	Relative afferent pupillary defect OS	IFN termined, IV hydration fluids, IVMP (3 days), oral prednisolone (2 weeks)	After 6 months visual acuity OS 0.1. Remaining visual field defect, relative afferent pupillary defect, optic disc pallor OS
44 year old man (13)	PEG-IFNα	2,5 months	Sudden painless inferior visual field defect OS	OS 20/80, OD 20/60. Visual field (inferior nerve fiber bundle) defect OU	OS: disc edema with splinter hemorrhages and cotton wool spots. OD: pallid edema of the optic disc with splinter hemorrhages and cotton wool spots	Relative afferent pupillary defect OU	IFN termined, IVMP (3 days), oral prednisolone (2 weeks)	After 2 months visual acuity OU 20/30. Remaining visual field defects. After 5 months optic disc pallor
33 year old man (16)	IFNα (9 MU/week) and RBV (800mg/day)	3 months (first undocumented episode after 2 months)	Sudden visual loss, headache	OS finger counting, OD hand movement	OD: optic disc swelling, tortuous dilated vessels, small scattered retinal hemorrhages	-	IFN termined	After 4 days visual acuity: OD finger counting, OS .0 1. Impairment of color vision OS. After 2 weeks vision 0.5 OD. No signs of retinopathy or color vision impairment
55 year old man (17)	PEG-IFNα (180 µg/week) and RBV (1000 mg/day)	5 months	Sudden bilateral visual loss	OS 0.2, OD 0.4	OU: disc edema	No response to visual evoked potentials OU	IFN termined, MP (5 days)	After 1 month visual acuity: OS 0.3, OD 0.5. Disc edema partially resolved. After 1 year visual acuity the same
28 year old man (18)	PEG-IFNα (135 µg/week)	2 months	Sudden, painless visual field defect OS	OS 20/80, OD 20/30. Visual field (inferior nerve fiber bundle) defect OU	OS: edema of the optic disc with splinter hemorrhages, cotton wool spots, OD: pallid edema of the optic disc with cotton wool spots	-	IFN termined, oral MP (32 mg/d 14 days, tapering off 2 months)	Visual disturbances resolved
57 year old man (19)	PEG-IFNα and RBV	6 months	Sudden, painless blurred vision OD	OD 20/60. Visual field (inferior altitudinal) defect, poor color vision OD	OD: edema of the optic disc with hemorrhage	Relative afferent pupillary defect OD. Decreased amplitude of visual evoked potentials	n.d.	After 6 weeks visual acuity OD 0. 1. Resolution of the optic disc edema

^a^ Abbreviations: CPM, cyclophosphamide; IVIg, intravenous immunoglobulin; IVMP, intravenous methylprednisolone; MTX, metothrexate; OD, oculus dexter; OS, oculus sinister; RBV, ribavirin

Considering the possibility of poor visual outcome associated with IFN alfa treatment, we recommend: examination of the eye fundus before treatment with PEG-IFN, especially of patients with diabetes and primary arterial hypertension, ophthalmologist consultation every 3 months during interferon treatment, if typical retinal changes are found (cotton wool spots and/or retinal hemorrhages), monitoring of visual acuity is recommended, in case of vision disorder and retinal lesion with the optic disc and macular edema, permanent discontinuation of antiviral drugs should be considered.
